# The Effect of Accelerator Dosage on Fresh Concrete Properties and on Interlayer Strength in Shotcrete 3D Printing

**DOI:** 10.3390/ma13020374

**Published:** 2020-01-14

**Authors:** Inka Dressler, Niklas Freund, Dirk Lowke

**Affiliations:** Institute of Building Materials, Concrete Construction and Fire Safety, TU Braunschweig, 38106 Braunschweig, Germany; n.freund@ibmb.tu-bs.de (N.F.); d.lowke@ibmb.tu-bs.de (D.L.)

**Keywords:** additive manufacturing, shotcrete 3D printing, interlayer strength, bond, accelerator

## Abstract

Recently, the progress in 3D concrete printing has developed enormously. However, for the techniques available, there is still a severe lack of knowledge of the functional interaction of processing technology, concrete rheology and admixture usage. For shotcrete 3D printing technology, we present the effect of accelerator dosages (0%, 2%, 4% and 6%) on fresh concrete properties and on interlayer strength. Therefore, early yield stress development up to 90 min is measured with penetration resistance measurements. Deformation of layers under loading is investigated with digital image correlation and a mechanical testing machine. One point in time (10 min after deposition) is examined to quantify vertical buildability of elements depending on the accelerator dosage. Four different interlayer times (0, 2, 5 and 30 min), which occur for the production of small and large elements as well as due to delay during production, are investigated mechanically as well as quantitatively with computed tomography regarding the formation of cold joints. With increased accelerator dosage, an instantaneous increase in early age yield stress and yield stress evolution was observed. An increase in interlayer time leads to a reduced strength. This is mainly attributed to the observed reduced mechanical interlocking effect of the strands. Finally, a model to describe interlayer quality is presented. In the end, advantages as well as limitations of the findings are discussed.

## 1. Introduction

3D printing is proclaimed to be “the third industrial revolution” [1], as many believe it has the potential to revolutionize all manufacturing processes. Even though cementitious building materials such as concrete can theoretically be used to create elements of any shape, in today’s concrete construction, simple shapes with a constant cross-section are largely used. This is due to the high cost of freeform formwork on the one hand and the low degree of automation in construction on the other hand. Compared to conventional production, 3D printing techniques hold an advantage for the fully automated production of components of high geometric complexity-even for small quantities. Therefore, 3D printing seems to be ideally suited for construction where piece production and geometric complexity are common [2]. CNC-based and robot-controlled 3D printing processes in particular offer the opportunity to minimize cost-intensive manual production. Freed from the constraints of conventional manufacturing, the shape of structural elements can be tailored to various requirements and functions. In contrast to traditional construction methods, particularly those used in concrete construction, where a single type of material is poured into formwork, it may also be possible to achieve functional grading (see, for example, [3]) by creating structural reinforced elements with non-standard geometries and varying the material properties during the printing process. Generally speaking, this would lead to more economic, higher-quality construction processes and more efficient material usage.

For large-scale additive manufacturing in construction, mainly extrusion or shotcrete 3D printing techniques, where the premixed cementitious material is extruded or sprayed in long strands, are applied. In general, two main strategies can be distinguished: (I) the deposition of narrow strands of several centimetres’ width, which are either stitched to create the desired structure or used to build a filigree formwork structure strengthened by an inner structure (compare concrete printing [4,5,6,7,8,9,10] and contour crafting [11,12,13,14]) and (II) the deposition of broad strands of several decimetres’ width, which are used to build the whole width of the component in a single pass as a kind of ‘infinite brick’ (compare, e.g., ConPrint3D [15,16]). The major advantage of these techniques is the high manufacturing speed for creating large-sized monolithic structures as well as the building size of the produced elements.

**Figure 1:**
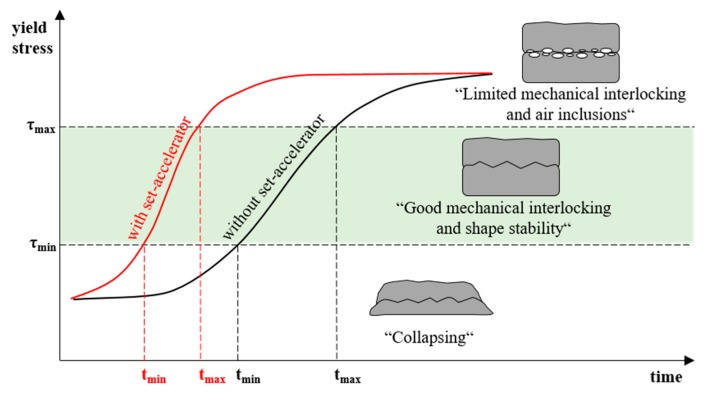
Principle of yield stress development over time with and without set-accelerator as well as the open window for processing shotcrete indicated by t_min_ and t_max._

High manufacturing speed means the need for a rapid structural build-up of the deposited material, as the stability of the printed strand is based on the self-supporting function of the concrete due to the absence of a supporting formwork. In addition to the inherent stability of the strand, the printed material also has to bear the load of the subsequent layers. To enable the printed layer to bear the load of the next layer without collapsing, a minimum yield stress is necessary [5,17,18,19,20]. For this purpose, t_min_ defines the point in time when a subsequent layer can be applied to the existing layer [20], see Figure 1. Achieving the minimum required yield stress is highly dependent on the mixtures composition and the used admixtures. In particular, the use of set accelerators significantly changes the setting behaviour of the concrete and enables, derived from this, the manufacturing of large building heights.

However, to make deposition techniques mechanically competitive for casting of concrete high interlayer adhesion, e.g., the prevention of cold joints, is required [21]. Therefore, the roughness of the interface region is relevant, which is predominantly determined by mixture composition (e.g., aggregate size, cement, water–cement-ratio, admixtures). Moreover, time delay between two layers, which often results from the production process itself, is important since limited interlocking occurs after a critical resting time [22]. For the continuous production of various sized elements, the nozzle will apply the adjacent layer within several seconds up to a few minutes. When production stop occurs, the adjacent layer will be applied several minutes up to a few hours later.

It is important to investigate the effect of yield stress development as well as the effect of different accelerator dosages on the quality of the layer interlocking. For this purpose, a maximum yield stress and, thus, a maximum delay time t_max_ need to be defined. This represents an upper limit with regard to the interlayer strength quality, Figure 1.

Although the progress in 3D concrete printing has developed enormously [23], there is still a severe lack of knowledge of the functional interaction of processing technology, concrete rheology and admixture usage. This paper presents for Shotcrete 3D Printing (SC3DP) technology the effect of a range of accelerator dosages (0%, 2%, 4%, 6%) on fresh concrete properties (yield stress evolution) and on interlayer strength.

## 2. Shotcrete 3D Printing (SC3DP) Technology

**Figure 2:**
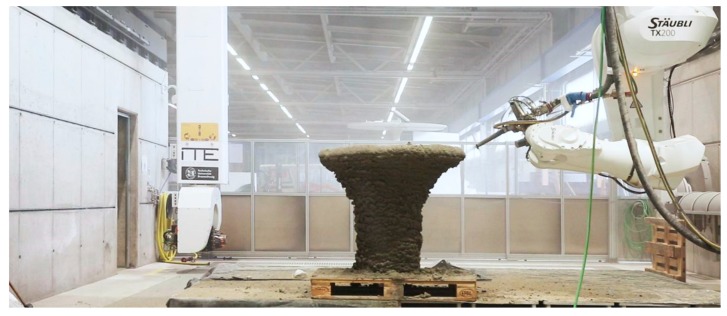
Digital Building Fabrication Laboratory (DBFL) at Technische Universität Braunschweig, Germany (© Institute of Structural Design Technische Universität Braunschweig), compare [25,29].

SC3DP is a novel additive manufacturing method using an automated wet-mix shotcrete process [24,25,26]. It is assigned to the open space deposition 3D printing technologies and is especially designed for the manufacturing of large-scale components, Figure 2**.** Due to the varying nozzle angle from 0° to 180°, full three-dimensional processing are achievable. The resulting geometry of the printed path can be varied by different process parameters such as nozzle distance and path velocity. Unlike the extrusion method, SC3DP uses high kinetic energy for the application of concrete. Specimens manufactured with this technology show an increase of contact surface between the layers, which allows a good interlocking effect [27]. For this reason, the use of high kinetic energy can lead to the minimization of cold joints [28]. However, due to the production without supporting formwork, the SC3DP as well as the extrusion process are based on a self-supporting of the printed material. The contrary requirements, which are caused by the pumping process and the required pumpability on the one hand and the subsequent rapid structural build-up as well as pursued interlayer strength on the other hand, necessitate the targeted use of admixtures (e.g., superplasticizer and set accelerator) [18,27]. Compressed air is applied in the nozzle to spray the pumped concrete. Moreover, admixtures may be induced in the nozzle, which are homogeneously distributed by the compressed air.

## 3. Effects on Interlayer Strength

It is commonly known that the bond between two layers in 3D-printing is a prerequisite to avoid weak spots and anisotropy (e.g., [21,22,30,31,32]). There are two main factors influencing the interface bond between two concrete layers [33]:

(a) mechanical effect: mechanical bonding relies solely on the physical attributes of the layers, e.g., micro roughness of substrate, cohesion and friction coefficients of the layers and age of the bottom strand as well as rheological properties of the single layers [31,32]. The inability of the layers to interlock lead to a reduced contact area and herewith to a reduced interlayer adhesion.
(b) chemical effect: chemical bonding occurs if the hydration and chemical bonding of particles occurs in between two layers [31].

Moreover, it is possible that there are further factors, such as homogeneity, affecting interface bond. However, these factors have not been systematically investigated yet.

Up to now, the effect of interlayer strength in the context of 3D printing has mainly been observed with extrusion-based techniques. **Anisotropic behaviour** was observed for mechanical strength of 3D-printed specimen [34,35,36,37], which is attributed to the oriented interface properties [38]. In [39], a flexural strength of a cement-based mortar with a water-to-binder-ratio of 0.42 for layers oriented perpendicular to a loading direction of 10.1 MPa was reported, whereas, for layers with parallel orientation, a reduced flexural strength of 5.3 MPa (approximately 48% strength loss) was determined. The anisotropic effect was found to be more distinct for tensile strength than for compressive strength, which indicates that the increase in interlayer strength could be due to the increase in contact surface, e.g., due to interlocking, of the layers [32]. The positive effect of an increase in contact area on interlayer strength has also been identified by [40,41].

A reduction of flexural strength with **interlayer time** in between the manufacturing of two layers was explained with a higher amount of pores/voids [41], microcracks at the interface as well as drying and plastic shrinkage [31,39]. In the latter, a reduction in flexural strength has been reported for specimen with a water-binder-ratio of 0.42 when the interlayer time in between the layers increased from one minute to ten minutes (up to 45% strength loss) or even one day (up to 85% strength loss), whereas, in [41], an interlayer strength reduction of 89% for a 10 minutes-interlayer time and 97% for 60 minutes-interlayer time was reported for a mortar with a water-cement-ratio of 0.36 for a printing speed of 3 cm/s. In addition, reference [9] stated that there is a time window for geopolymer mortars in which the interval time has negligible effect on interlayer strength, whereas, outside of the window, the negative effects are significant. In [42], it is found that an increase in **water–cement ratio** has a positive effect on relative interlayer strength for an interlayer time of two hours. For a specimen with a water–cement ratio of 0.40, the relative interlayer strength was reported to be nearly 100% (compared to monolithic specimen), whereas it dropped to below 60% for a specimen with a water–cement ratio of 0.20. Therefore, extremely localized drying is assumed to be the origin of a drop in bond strength in particular for flowable mortars with low water-to-cement ratios since evaporated liquid is not replaced quickly enough by liquid from the inner part of the layer. Herewith, a weak region with less hydrated cement than in the rest of the layer is prevalent. This could be complemented by the finding that moisture content of the bottom layer has an effect on interlayer strength, where a higher moisture content led to an increase in interlayer strength within the investigated boundary conditions [30,43].

The usage of silica fume in **mixture composition** (positive effect) [44], decrease in aggregate size and aggregate-to-cement ratio (positive effect) [45] were found to affect interlayer strength. In addition, a high structuration rate of the material is negatively affecting interlayer strength of geopolymer mortars [9]. In [46], it is stated in the context of distinct-layer casting of self compacting concrete that the thixotropic behaviour should remain under a certain threshold value to allow subsequent layers to mix. This means that there is a critical timeframe for each mixture composition, which is influenced by factors such as particle packing, cement type [47], or admixtures [48], in order to enable a high interlayer bond.

In addition to this, the **process of applying the layer** has been identified to affect interlayer strength. In [34], it is stated that there is no significant effect on interlayer strength of 3D printed concrete when the distance from the nozzle to layer is changed. By contrast, [49] found an increase of the nozzle height to have a negative effect on interlayer strength of geopolymers mortars. This is supported by [9], who measured the same correlation and found additionally the mixture composition as an influencing factor when varying the nozzle distance. Reference [41] investigated the effect of printing speed on interlayer strength. They measured a strength reduction of approximately 50% when increasing the printing speed from 1.7 cm/s to 3 cm/s and explained the effect with an increase in voids and a decrease in surface roughness of the contact area in between the layers. In [28], the interlayer adhesion is compared for extrusion- and SC3DP-technique. For the same material, the mechanical strength (expressed in flexural and compressive strength) of the SC3DP-specimen is higher than that of extruded specimen. This is assumed to be due to higher induced kinetic energy, which leads to a reduced air void content (expressed in a higher density) and herewith higher strength.

The findings regarding the effects on interlayer strength are not always consistent—compare [23]. In addition, there are nearly no data available on systems with significant structural build-up as it is prevalent in SC3DP-technology. The development of fundamental theories is necessary to explain the observed effects regardless of a specific mixture composition. Moreover, coherent testing procedures regarding the production of the specimen (e.g., geometry of specimen, loading direction) need to be established to make results comparable in the future.

## 4. Materials and Methods

### 4.1. Scope and Concept of Experimental Investigation

The presented investigations pursued the goal to determine the effect of accelerator dosage and the time gap in between two strands of shotcrete on (a) vertical buildability and (b) interlayer strength, e.g., mechanical performance. Both accelerator dosage and interlayer time are assumed to have an effect on the properties of the contact area in between the layers.

In line with this, a multi-scale approach is chosen for the investigations of shotcrete layers with variations in the content of accelerator (0%, 2%, 4%, 6%) and interlayer time in between two strands (0 min, 2 min, 5 min, 30 min). On a macroscopic level, the fresh properties (yield stress evolution over time, deformation under loading, geometry) as well as hardened properties (compressive and 3-point-bending-tests) are investigated. On a microscopic level, the interlayer zone is investigated in more detail on drilling cores using micro-computed-tomography (µCT) and image post-processing.

**Figure 3:**
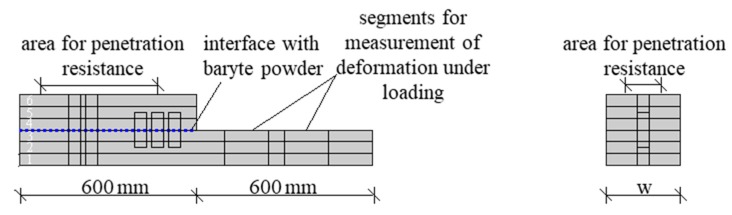
Principle of sampling: production of three long layers and subsequently three short layers.

The manufacturing process is divided into two steps. In the first step, three layers are sprayed over a length of 1200 mm, which corresponds with an approximate sample height of 10 cm. For mechanical investigations on the printed layers in fresh state, one half of the sample was removed after completion and transported to the testing machine for deformation measurements under loading. The remaining half of the sample was left in the working space of the printer. After a designated interlayer time ∆t (0 min, 2 min, 5 min, 30 min), three additional layers are sprayed on the remaining 600 mm as a second manufacturing step. A total sample height of approximately 200 mm and a width w of approximately 125 mm is targeted. On the sixth strand, the yield stress measurements with a penetrometer are performed. An overview of the principle of sampling is shown in Figure 3.

The series of investigation are named as indicated in Table 1. Every experiment reveals the accelerator dosage in percent and-if relevant-the interlayer time ∆t in between layer three and four in minutes. Each experiment is repeated twice.

### 4.2. Materials and Mixture Preparation

The material used in this series of experiments consists of an Ordinary Portland Cement (OPC, CEM I 52.5) and a quartz sand with a maximum grain size of 3.15 mm. A detailed overview of all used components and chemical admixtures is given in Table 2.

For the experiment, batches of 75 L are prepared in a pug pill mixer (Mader WM Jetmix 125/180, Erbach, Germany). The regime in the compulsory mixer is basically divided into two phases:

- Water was put into the mixer. For 2 min, all components are added continuously into the mixing container while the mixing tool was rotating. The order of addition was set as follows: a dry premixed mixture of cement, ground limestone and stabilizer is added, followed by the dry premixed aggregates. Moreover, superplasticizer was added constantly over the entire 2 min.
- Continuous mixing for a further 2 min.

### 4.3. Methods

#### 4.3.1. Material Processing and Test Specimens

**Figure 4:**
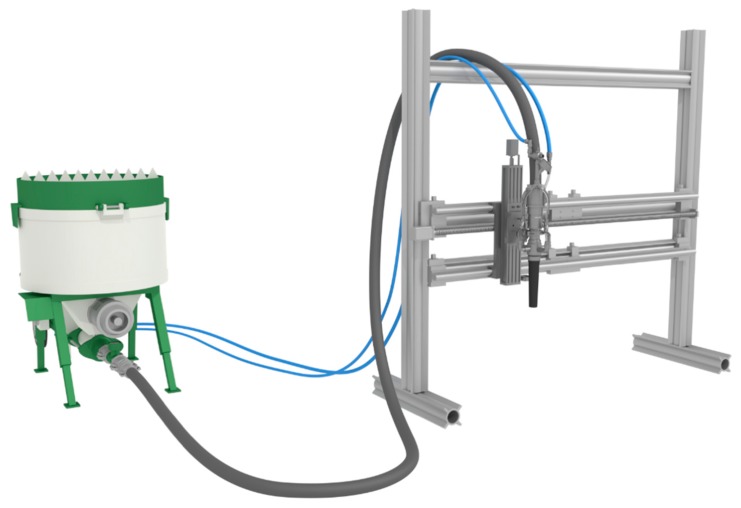
Experimental setup for the Smart Additive Manufacturing Material Investigator (SAMMI) for Shotcrete 3D-printing (SC3DP) experiments. *x*–*z*-linear axis with a nozzle.

For material processing, a small lab-scale SC3DP unit is used (Smart Additive Manufacturing Material Investigator—SAMMI, manufactured inhouse). SAMMI contains an *x*–*z*-linear axis with maximum gantry speed of up to 5 m/min, Figure 4. The shotcrete material is produced outside of the working space with a pug mill mixer and pumped (Mader WM Variojet FU, Erbach, Germany) through a hose to a shotcrete nozzle. Inside the nozzle, the concrete is torn up by air twice—firstly with pressurized air only and secondly with pressurized air and-if applicable - accelerator, compare also [50].

The parameters for the experiments at hand are as follows:

Discharge rate of concrete pump: 0.8 m^3^/h,
Hose: 5 m, diameter: 35 mm,
Volume air flow: 45 m^3^/h,
Working distance (nozzle to specimen): 20 cm,
Gantry speed: 4.5 m/min.

After being manufactured, all specimens are stored in an air-conditioned environment at 20 °C and 65% relative humidity until the investigation of mechanical properties.

#### 4.3.2. Investigations on Fresh Properties

The consistency of fresh concrete properties is measured and documented by the slump test (according to DIN EN 12350-2 [51]; cone dimensions: height = 300 mm, bottom diameter = 200 mm, top diameter = 100 mm). This test examines the vertical slump of the concrete by filling it into a slump cone and removing it. For quality control, a sample is taken from each mixed batch before filling the pump.

A handheld shotcrete penetrometer (Mecmesin, West Sussex, UK) is used to discontinuously investigate the penetration resistance evolution of fresh concrete from 5 to 90 min after deposition. The penetrometer needle used has a diameter of 3 mm, a cylindrical height of 12.5 mm and a cone height of 2.5 mm. According to [52,53], penetration resistance is correlated to yield stress of cement paste and mortar when a sufficiently large needle is used. Therefore, the yield stress is calculated according to [52]. For each point in time, five repetitions are conducted in the centre of the specimen.

The height of every layer is measured with a ruler after deposition. Moreover, the width is determined and used to calculate the stress in the specimen under loading.

To quantify the shape stability of the manufactured strands, two 30 cm long segments are used, Figure 3. These segments, consisting of three layers each, are loaded in a testing machine (Zwick/Roell Z020, Ulm, Germany) after a defined time period of t_1_ = 10 min after deposition with a testing speed of 2 N/s up to a force of 1000 N to simulate the additional load when applying 50 additional layers. The load is applied to the specimen over a length of 20 cm and can be recalculated to stress over the loaded width of the specimen. The selected settings of the load application are representative for a typical printing progress of a wall with a height of approximately 1.5 m within 10 min. In order to ensure a constant load introduction, each specimen was loaded with a preload of approximate 40 N. The deformations of the printed strands are quantified by an optical measuring system (Aramis, GOM, Braunschweig, Germany). This system is composed of two cameras, which record the deformation on the basis of previously defined reference points (pixel length = 142 µm) [54]. Reference points were attached to the testing machine (for validation, GOM, Braunschweig, Germany) and inserted on sticks into the layer interface during the production process, Figure 7a. Thus, information on the deformation of the individual layers can be obtained in addition to the overall deformation.

#### 4.3.3. Investigations on Mechanical Properties

In order to examine mechanical properties prisms perpendicular to the layer direction are cut. The prisms were cut approximately 3 weeks after production. In order to minimize the impact of cutting, a fine saw blade was used. Since a prism length of 160 mm with a central Δt-interface could not be realized for every sample, the side-length ratio was scaled to a length of 125 mm (20 × 20 × 125 mm^3^). The prisms are tested after 28 days of standardized storage in a 3-point bending test. The previously created ∆t-layers were positioned in the middle so that the highest bending moment was applied at this interface. After finishing the bending tests, the remaining halves of the specimens are tested for compressive strength. In order to obtain a comparison to monolithic samples for all accelerator dosages (0%, 2%, 4%, 6%), material is removed from the nozzle during the tests and subsequently cast into prism moulds. The conventionally produced samples are stored together with the printed specimens under standardized conditions (20 °C, 65% r.H.) and are tested after 28 days.

#### 4.3.4. Investigations on Interlayer Characteristics

**Figure 5:**
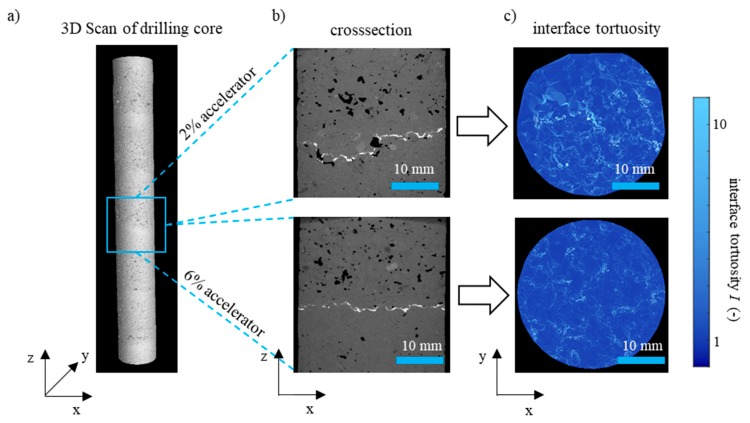
Method to investigate the interlayer exemplified for two experiments with various content in accelerator: (**a**) 3D scan of a drilling core, (**b**) interlayer between strand three and four (white line) and (**c**) calculation of the interface tortuosity.

To investigate the interface between two layers on a microscopic scale, drilling cores (diameter 30 mm) are taken from the specimen. Since baryte powder is placed in between layers 3 and 4 during production of the layers, the interface with baryte powder can be examined in more detail [55]. The specimen is placed into a μCT and 3D scanned (GE phoenix, Boston, MA, USA, voltage: 160 kV, current: 230 µA, number of images: 1000, image average: 3, filter: 0.1 mm Cu, exposure: 500 ms for all specimen, voxel edge length: 33.7 µm). From the X-ray projections of each specimen, the volumetric image is obtained by applying a 3D reconstruction algorithm with the software phoenix datos|x2 (GE Sensing & Inspection Technologies, Boston, MA, USA). The software VG studiomax 2.2 (Volume Graphics, Heidelberg, Germany) is used to convert the reconstruction to RAW-files. Matlab 2018b (Mathworks, Natick, MA, USA) is used for postprocessing of the RAW-files with a customized script. The script segments the baryte powder, which represents the interlayer, with a threshold approach. Then, the interlayer is reduced to one scalar height value *z* at every coordinate (*x,y*) along the base area. Therefore, the thickness is reduced to one voxel by using its median *z*-coordinate as the boundary position. Then, the segmented discrete points are bilinearly interpolated via a mesh and the generated data are saved in a 2D array. Then, further analysis of the interlayer is performed, Figure 5. Here, the (interpolated) real contact surface area *A_c_* (mm^2^) is divided by the basic surface area *A*_0_ (mm^2^) to quantify the local effective interlayer in the 2D array with an interface tortuosity *I* (-):

(1) $$I=\frac{A_{c}}{A_{0}}$$

The mean interface tortuosity *I* (-) is calculated for the middle interface of each drilling core according to Equation (1). The lower the coefficient, the weaker the interlocking effect. If the mean interface tortuosity approaches 1, there is no mechanical interlocking in between two layers. This factor is mathematically similar to a 3D-tortuosity, which is usually used to describe the structure of cracks [56].

Furthermore, the micro roughness $R_{a}$ of a printed layer (before applying the subsequent layer) was determined on the top layers of specimen a0 to a6 by means of a digital microscope (Keyence, VHX-2000D, magnification 30 × 20) since in literature micro roughness was found to affect interlayer strength, compare [41]. Therefore, a profile in the printing direction is taken from two specimens per accelerator dosage with a total measurement distance *l* of 120 mm. Then, the mean vertical deviation *Z*(*x*) of the surface from an ideal smooth surface is determined according to DIN EN ISO 4287 [57]:

(2) $$R_{a}=\frac{1}{l}\int_{0}^{l}\left|Z\left(x\right)\right|dx$$

## 5. Results and Discussion

### 5.1. Fresh Properties

The slump tests carried out as part of quality control give sufficient uniform values. The average slump was measured to be 23 mm with a standard deviation of 5 mm. According to DIN EN 12350-2 [51], the used material can be classified into the category S1 (plastic concrete).

**Figure 6:**
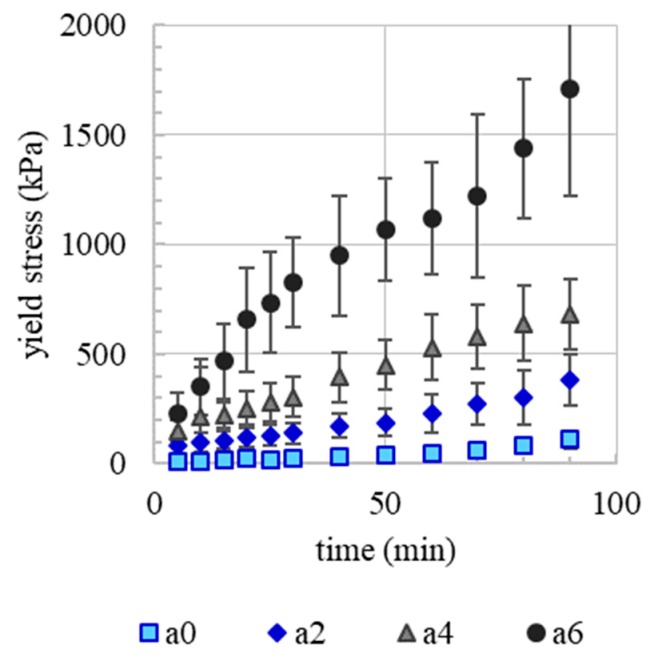
Yield stress evolution over time after deposition of sprayed strands containing 0% (a0), 2% (a2), 4% (a4) and 6% (a6) of the accelerator.

An increase in yield stress over time is observed for all sprayed strands, Figure 6 As expected, an increase in accelerator dosage leads to an increase of initial yield stress as well as slope of the yield stress evolution. After 90 min, the yield stress of a6 (6% accelerator, 1710.4 kPa) is approximately 15 times higher than the yield stress of the sprayed layers without accelerator (a0, 110.9 kPa). Obviously, the (alkali-free) accelerator provides rapid stiffening of the concrete, as it is desired for SC3DP to enable a fast vertical building progress. This is mainly attributed to aluminium sulphate, which reacts with calcium and produces ettringite and aluminium hydroxide [58,59]. Besides the general effect of accelerating admixtures, spraying could enhance accelerator reactivity and C_3_A hydration [60] and could lead herewith to a faster yield stress evolution in early ages compared to extruded material. Moreover, yield stress evolution might be affected by evaporation from the specimen’s surface.

The results of the loading tests show a clear correlation between the accelerator dosage and the deformation of the printed layers. For investigating the transfer of force between two concrete layers, the focus was placed on the lowest interlayer (between strands 1 and 2), which is the layer with the highest load in real components. For an accelerator dosage of 0%, the applied load of 1000 N, which is approximately equivalent to manufacturing process of a 1.5 m high wall within 10 min, results in a deformation of 2.9 mm. By using the 2% accelerator, the maximum displacement decreased by more than 60% to 1.2 mm. A further increase in the accelerator dosage to 4% and 6% is accompanied by a further decrease in total settlement to 0.8 and 0.5 mm, respectively. Table 3 gives a summary of the measured settlements for a loading of 1000 N.

**Figure 7:**
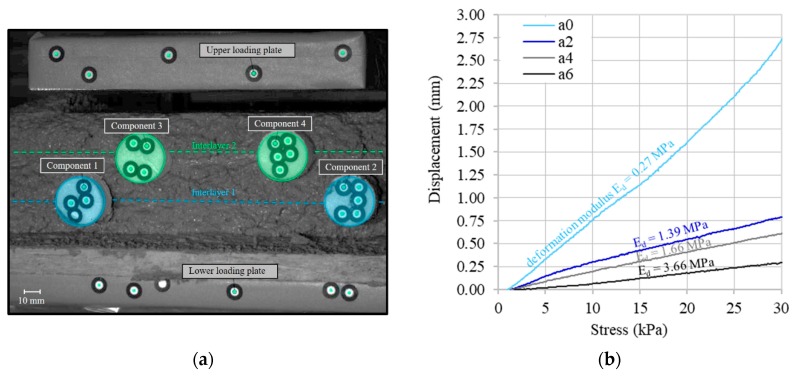
Optical measurement of interlayer displacement; Specimen with inserted reference components (**a**), Displacement–stress curve and deformation modulus of the first interlayer for a0 to a6 10 min after deposition (**b**).

Since the loaded samples have process related varying widths, the force increase is converted into a stress increase via the measured specimen geometry. The previously described effect of accelerator dosage on deformation can thus be converted into an effect on deformation modulus E_d_ (slope of the stress–strain curve) of the fresh concrete, Figure 7b. Accordingly, the results show an increase of deformation modulus by a factor of approx. 14 from a0 (unaccelerated, 0.27 MPa) to a6 (6% accelerator, 3.66 MPa). Particularly clear is the step from a0 to a2, which is accompanied by a displacement difference of about 2 mm at 30 kPa. Therefore, it can be stated that rather low accelerator dosages lead to a significant reduction in material deformation. Thus, a faster vertical building progress is achievable.

In the presented experiments, the point in time when a subsequent layer is applicable to an existing layer without collapsing (t_min_) is always exceeded. However, it is assumed that this point in time is highly dependent on the individually defined maximum permissible displacements of the produced element.

### 5.2. Interlayer Characteristics and Mechanical Properties

The results of the compressive strength tests show no noticeable correlation between the set-accelerator dosage and the resulting 28d-compressive strength of 3D-printed specimens, Table 4.

**Figure 8:**
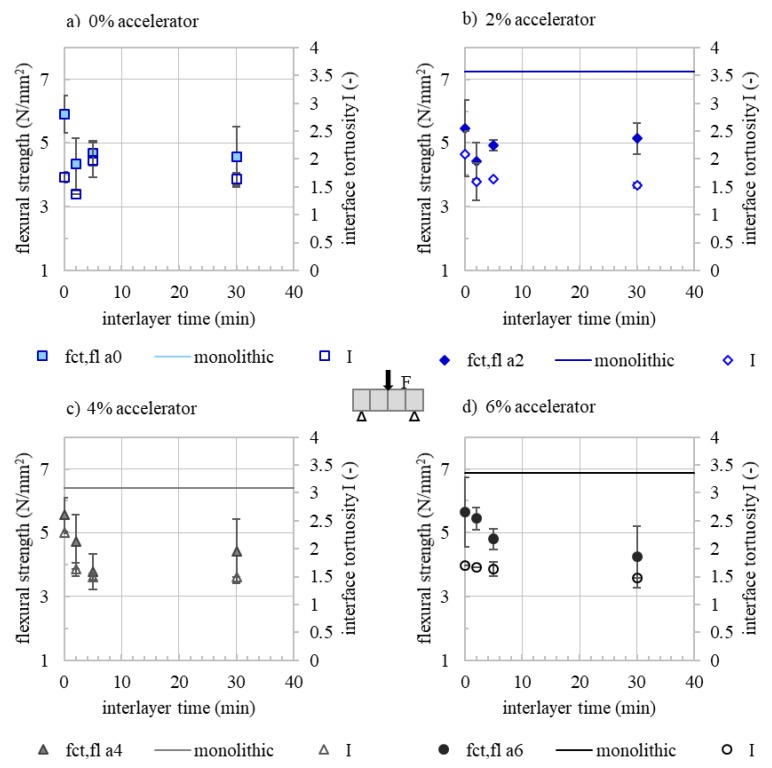
28 d flexural strength f_ct_ of monolithic specimen and specimen with various interlayer times as well as according interface tortuosity I of (**a**) a 0%, (**b**) a 2%, (**c**) a 4% and (**d**) a 6% (for each accelerator dosage, a minimum of six drilling cores and seven prisms is evaluated) depending on the interlayer time.

The results of the effect of interlayer time on flexural strength and interface tortuosity for sprayed concrete a0 to a6 as well as the flexural strength of conventional casted specimens are shown in Figure 8. First of all, one can see that flexural strength of the accelerated specimens is reduced by 0.9 to 1.8 N/mm^2^, when the material is applied by SC3DP (with interlayer time 0 min) compared to the conventional casted specimens (compacted via poking, indicated with a solid line). The flexural strength of the sprayed specimen (solid symbols), where the load is applied parallel to the layer orientation, characterises the interlayer quality. With an interlayer time of more than 0 min, a reduction of flexural strength of 3D printed specimen, i.e., a reduction of interlayer quality, is observed. Interestingly, the decrease is particularly pronounced with short interlayer times (0–5 min). With a long interlayer time of 30 min, there is no further significant reduction of flexural strength. For example, for a0t5, a reduction in flexural strength of 21% (1.2 N/mm^2^) and for a0t30 a reduction of 22% (1.2 N/mm^3^) is observed compared to a0t0. A similar behaviour has been observed for the extrusion process (compare Section 3). With regard to a practical application, this means that interlayer times of less than 2 min should be envisaged for path planning in order to avoid a significant (more than 20%) loss in interlayer strength. However, from the foregoing, a maximum interlayer time (t_max_), which represents an upper limit with regard to the interlayer strength quality, is not distinctively assignable. This point in time is highly dependent on the individually defined maximum sufferable reduction in interlayer strength.

**Figure 9:**
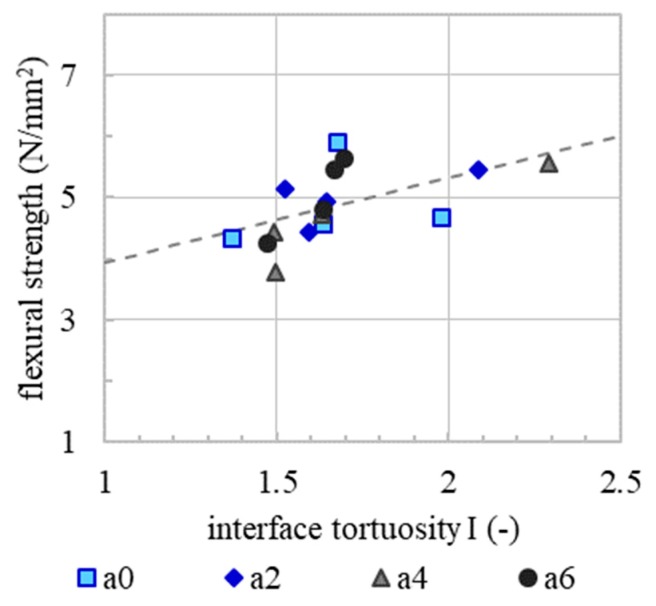
Linear correlation (dashed line) of mean flexural strength and mean interface tortuosity of drilling cores from the printed specimen.

To understand the underlying mechanisms of the observed behaviour of interlayer strength over time, the interface tortuosity of the drilling cores was determined, Figure 8 (empty symbols). The mean interface tortuosity of the drilling cores ranges from *I* = 1.37 to 2.29 (-). As with flexural strength (filled symbols), the interface tortuosity tends to decrease as interlayer time increases. The lower the initial yield stress of the layer, the more deformation can take place and thus higher interlayer tortuosity can be achieved. With increasing accelerator dosages and increasing interlayer times, the initial yield stress of the underlying layer increases. As a result, a lower interlayer tortuosity and herewith a lower interlayer quality is generated. Therefore, measured flexural strength on the specimen correlates with the interface tortuosity, Figure 9.

**Figure 10:**
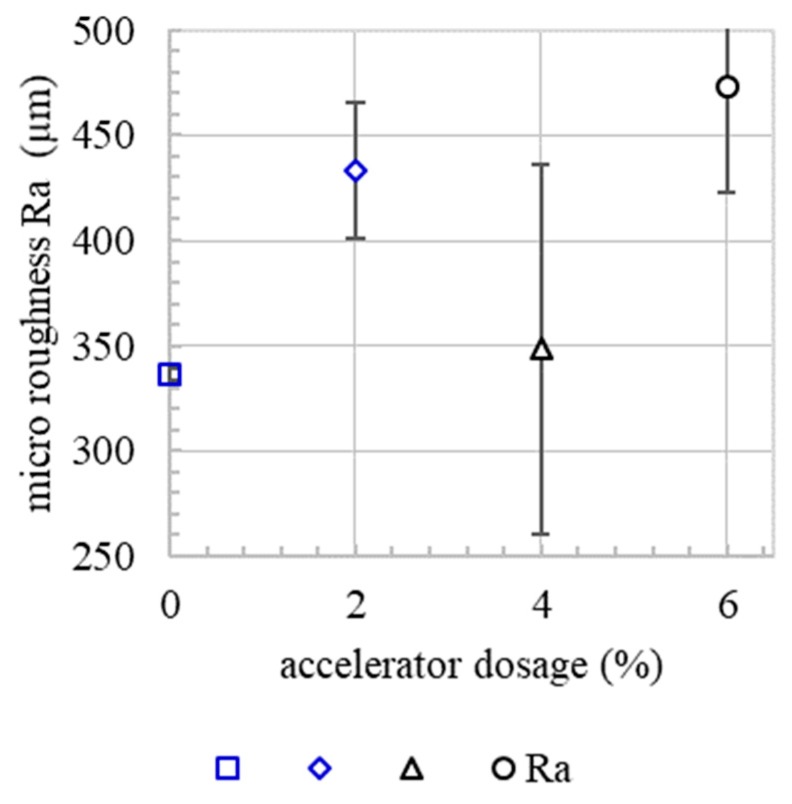
Micro roughness of the surface layer of specimen containing 0% (a0), 2% (a2), 4% (a4) and 6% (a6) of accelerator.

**Figure 11:**
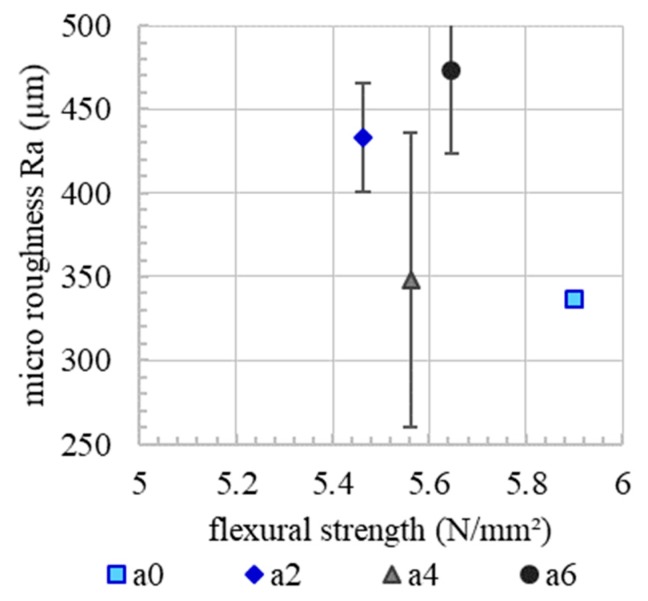
No correlation between initial micro roughness and flexural strength of an interlayer time of 0 min.

**Figure 12:**
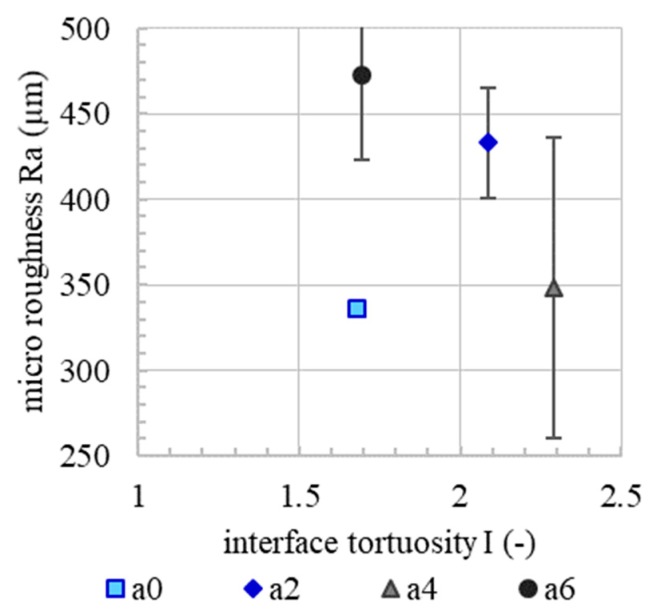
No correlation between initial micro roughness and interface tortuosity for an interlayer time of 0 min.

**Figure 13:**
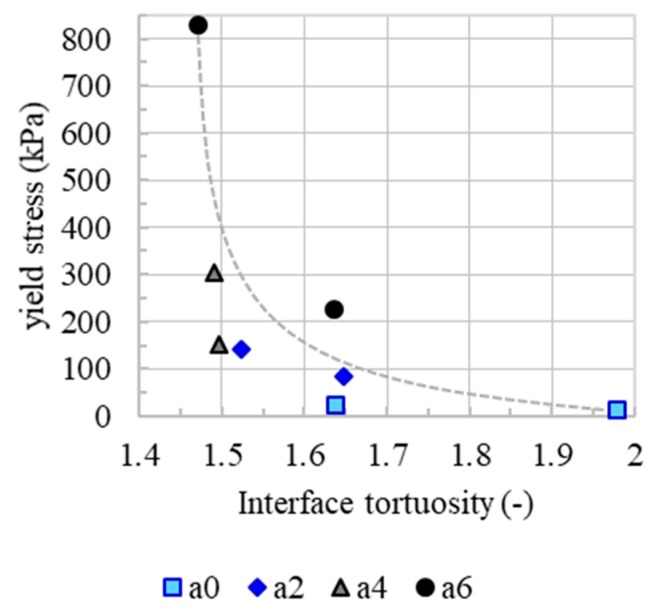
Reduction of Interface tortuosity with increasing yield stress. Exemplary shown for the specimen a0–a6 with interlayer time 5 and 30 min.

Since micro roughness tends to increase with increasing accelerator dosage, Figure 10. Since the basic mixture, e.g., the granulometric characteristic, is the same for all experiments, it is presumed that the micro roughness correlates with the yield stress of the material. In the spraying process, the material volume receives the same kinetic energy. However, material with a higher content of accelerator needs a higher energy to reach the same level of compaction as a material with a lower content of accelerator. This leads to a rougher surface. However, there is no correlation between the micro roughness and flexural strength or the interface tortuosity, Figure 11 and Figure 12. This means that, in the SC3DP process, the mechanical bond between two layers is induced less by the micro roughness generated on the free surface during the production of the bottom layer than by the tortuosity generated by the imprinting of particles during the application of the subsequent layer. Here, micro roughness plays a minor role regarding interlayer strength compared to interface tortuosity, which depicts the macro roughness of the interlayer and corresponds with a vertical interlocking-amplitude of up to 4.3 mm. The interlocking effect of interface tortuosity is several magnitudes higher than micro roughness. The characteristic of the interlocking is depending on the yield stress of the lower layer (deformability, which is time-dependent) and has therefore a superior effect on the flexural strength for SC3DP specimen, Figure 13.

### 5.3. Modeling Interlayer Strength

In view of the above considerations, it should be possible to quantitatively describe the flexural strength of the SC3DP specimen with dependence on interface tortuosity, which encompasses all relevant boundary conditions. Therefore, a mechanical model is proposed based on an approach of Walraven [61] for the stress transmission in cracks in the direction of crack:

(3) $$\sigma =\sigma_{pu}\left(A_{x}+\mu \cdot A_{y}\right),$$

(4) $$\mathrm{with}\;\sigma_{pu}=5.83f_{cc}^{0.63}$$

Here, $\sigma_{pu}$ (N/mm^2^) is the yielding strength of the matrix depending on uniaxial compressive strength of concrete $f_{cc}$ (N/mm^2^), $A_{x}$ (-) and $A_{y}$ (-) are the projected contact areas for a crack in *x*- and *y*-directions, *µ* = 0.5 (-) is the coefficient of friction.

Based on Equations (3) and (4), the model is adopted to the case at hand. The interlayer is considered as weakened area and is therefore the relevant location for stress transmission during flexural strength tests. Therefore, stress σ is replaced by flexural strength $f_{ct}$ (N/mm^2^). Moreover, the projected contact areas are-due to isotropy reasons-replaced by the interface tortuosity I (-) between two layers. A dimensionless coefficient ε (-) is used as model shift factor between crack–stress transmission and determination of interlayer quality:

(5) $$f_{ct}=\sigma_{pu}\cdot I\cdot \varepsilon$$

**Figure 14:**
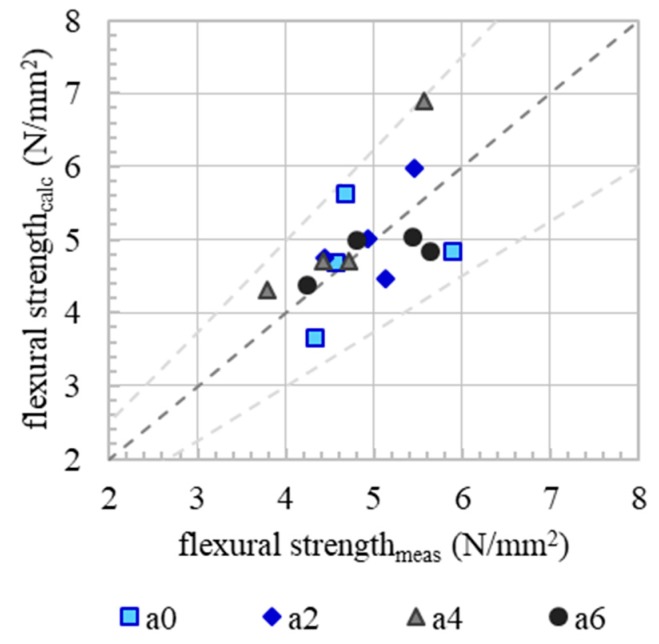
Correlation (dark grey dashed line) of measured and calculated flexural strength. Calculations are based on a modification of Walraven’s model for stress transmission in cracks. Bright grey dashed line indicates the range of certitude (0.75× and 1.25×).

It can be shown that a good agreement exists between the model according to Equation (5) when using a model shift factor ε= 0.037, Figure 14.

From the above, it is concluded that the macro roughness of the interlayer, expressed as interface tortuosity *I*, is a key parameter to describe interlayer quality. The interface tortuosity is affected by yield stress and herewith accelerator dosage and point in time of application of the subsequent layer. Moreover, the compressive strength is relevant, which is interrelated to flexural strength of concrete.

## 6. Conclusions and Outlook

This contribution focusses on the effect of accelerator dosage and interlayer time on fresh concrete properties and interlayer strength in Shotcrete 3D Printing (SC3DP). In the experimental investigations, the accelerator dosage is varied from 0% to 6%. A severe increase in yield stress after deposition and in yield stress evolution over time is observed when adding accelerator. This enables a higher vertical building rate, which is highly relevant for practical applications. By using a 2% accelerator, the deformation in the lowest layer is reduced significantly from 2.7 mm to 0.8 mm compared to the unaccelerated material for an applied stress of 30 kPa. By using a 6% accelerator, the deformation of the lowest layer is even reduced to 0.3 mm. By calculating the deformation modulus E_d_, the effect of accelerator dosage on the mechanical properties of fresh concrete could be quantified. For material with 6% accelerator compared to a 0% accelerator, the deformation modulus is about 14 times higher.

An increase in accelerator dosage leads to an increase in yield stress evolution. A clear correlation of yield stress and resulting interlayer strengths is found. In order to quantify and explain this effect, mechanical performance tests as well as additional µCT-images of the interlayer zones are carried out. Since various time spans may occur before the application of a subsequent layer, four interlayer times (0, 2, 5 and 30 min) are examined. Based on the computed tomography results, an interface tortuosity, which is a parameter describing the interlocking of the layer’s interface, could be determined for each setup. Independent from the accelerator dosage, the largest decrease in interlayer strength is observed for interlayer times between 0 and 5 min. For longer interlayer times (5 to 30 min), no further significant reduction of interlayer strength is measured. With regard to a practical application, an interlayer time of less than 2 min is recommended, which needs to be considered e.g., for path planning in order to avoid a significant loss in interlayer strength. By including the analysis of µCT-results, a correlation between decreasing interlayer strength and decreasing interlayer tortuosity is noticed. Thus, it can be stated that, in SC3DP, the interlayer strength is significantly influenced by interlayer tortuosity. Varying interlayer tortuosity is deduced to the initial yield stress of the underlying layer to which a subsequent layer is applied. Interlayer tortuosity is higher and herewith a higher interlayer quality is generated, when short interlayer times are prevalent.

Finally, to quantify the overall mechanical performance of SC3DP elements, a model is developed depending on compressive strength and interface tortuosity. These findings can be used to improve interlayer strength in the future, e.g., by purposefully manipulating the interlayer roughness by process parameters. In this particular field, further studies are envisaged.

## Figures and Tables

**Table 1:** Overview of the experiments with variations in accelerator dosage (a) and interlayer time (t).

Accelerator Dosage	Interlayer Time			
	0. Min	2. Min	5. Min	30. Min
**0% Accelerator**	a0t0	a0t2	a0t5	a0t30
**2% Accelerator**	a2t0	a2t2	a2t5	a2t30
**4% Accelerator**	a4t0	a4t2	a4t5	a4t30
**6% Accelerator**	a6t0	a6t2	a6t5	a6t30

**Table 2:** Mixture compositions of used mortar.

Components	Value	Unit
Portland Cement (CEM I 52.5)	600	kg/m^3^
Ground limestone	97	kg/m^3^
Aggregate, d = 0–3.15 mm	1258	kg/m^3^
Water	270	kg/m^3^
Stabilizer	0.1	M.-%
PCE superplasticizer	0.3	M.-%/cement
Alkali-free set accelerator	0–6	M.-%/cement

**Table 3:** Total deformation of lowest layer for a loading of 1000 N (equal to 50 additional layers or a wall with a height of approximately 1.5 m) 10 min after deposition.

Accelerator Dosage	Total Deformation of Lowest Layer at a Load of 1000 N
**0%**	2.9 mm
**2%**	1.2 mm
**4%**	0.8 mm
**6%**	0.5 mm

**Table 4:** 28d compressive strength f_c_ of 3D-printed specimens.

Accelerator Dosage	Averaged Compressive Strength f_c_	Standard Deviation σ_fc_
**0%**	59.9 N/mm^2^	3.4 N/mm^2^
**2%**	64.8 N/mm^2^	2.8 N/mm^2^
**4%**	66.0 N/mm^2^	4.6 N/mm^2^
**6%**	65.7 N/mm^2^	3.0 N/mm^2^
